# Presentation and outcomes of indigenous Australians with peripheral artery disease

**DOI:** 10.1186/s12872-018-0835-z

**Published:** 2018-05-16

**Authors:** Tejas P. Singh, Joseph V. Moxon, Genevieve N. Healy, Yvonne Cadet-James, Jonathan Golledge

**Affiliations:** 1 Queensland Research Centre for Peripheral Vascular Disease, College of Medicine and Dentistry, James Cook University, Townsville, QLD 4811 Australia; 2 The Department of Vascular and Endovascular Surgery, The Townsville Hospital, Townsville, QLD Australia; 3 The University of Queensland, School of Public Health, Herston, QLD Australia; 4 Indigenous Centre, James Cook University, Townsville, QLD Australia

## Abstract

**Background:**

The risk factors for peripheral artery disease (PAD) are more common in Indigenous than non-Indigenous Australians, however the presentation and outcome of PAD in Indigenous Australians has not been previously investigated. The aim of this prospective cohort study was to compare the presenting characteristics and clinical outcome of Indigenous and non-Indigenous Australians with PAD.

**Methods:**

PAD patients were prospectively recruited and followed-up since 2003 from an outpatient vascular clinic in Townsville, Australia. Presenting symptoms and risk factors in Indigenous and non-Indigenous patients were compared using Pearson’s χ2 test and Mann Whitney U test. Kaplan Meier survival analysis and Cox proportional hazard analysis were used to compare the incidence of myocardial infarction (MI), stroke or death (major cardiovascular events) among Indigenous and non-Indigenous patients.

**Results:**

Four hundred and one PAD patients were recruited, of which 16 were Indigenous and 385 were non-Indigenous Australians. Indigenous Australians were younger at entry (median age 63.3 [54.7–67.8] vs 69.6 [63.3–75.4]), more commonly current smokers (56.3% vs 31.4%), and more frequently had insulin-treated diabetes (18.8% vs 5.2%). During a median follow-up of 2.5 years, five and 45 major cardiovascular events were recorded amongst Indigenous and non-Indigenous Australians, respectively. Indigenous Australians were at ~ 5-fold greater risk of major cardiovascular events (adjusted hazard ratio 4.72 [95% confidence intervals 1.41–15.78], *p* = 0.012) compared to non-Indigenous Australians.

**Conclusions:**

These findings suggest that Indigenous Australians with PAD present at a younger age, have higher rates of smoking and insulin-treated diabetes, and poorer clinical outcomes compared to non-Indigenous Australians.

## Background

Peripheral artery disease (PAD) refers to a range of stenosing and aneurysmal disorders of the extra-coronary and extra-cerebral arteries [1]. Examples of common PAD pathologies include lower limb occlusive disease, abdominal aortic aneurysm (AAA), and carotid artery stenosis. The burden of PAD is significant. Lower limb occlusive disease is estimated to affect ~ 200 million people worldwide, [2] and ~ 20 million people are estimated to have AAA [3]. Moreover, the prevalence of PAD is expected to double by 2040 due to population aging [4]. Despite current best care, PAD patients are at approximately three times higher risk of cardiovascular and all-cause mortality, compared to those without PAD [5]. Mortality attributed to PAD has been estimated to be higher in Australasia than in any other part of the world (3.89 per 100, 000 [95% CI 2.2–6.9]) [6]. The reasons for this have not been clearly demonstrated although one possible explanation is the high incidence and complication rates of PAD amongst sub-sets of the Australasian population. Indigenous Australians could contribute to such a high risk sub-set since they have previously been reported to have an excess of risk factors for PAD and its complications [7].

Indigenous Australians (Aboriginal and Torres Strait Islanders) have been reported to have a life expectancy of approximately 17 years less than non-Indigenous Australians, and a burden of disease 2.5 fold higher [8]. Non-communicable diseases represent 70% of the health gap, of which cardiovascular diseases are the leading contributor, followed by diabetes [9]. Despite awareness of the excess burden of cardiovascular diseases in Indigenous Australians, it has been difficult to establish health priorities and policy for many vascular conditions. A major reason for this is the lack of reliable clinical data, as currently most data available stems from administrative government data, which does not capture the complete spectrum of diseases and associated risk factors. This is particularly true for medical conditions which require specific investigation based diagnosis such as PAD.

Indigenous Australians have previously been reported to have a 3-fold higher risk of developing PAD (adjusted for age, diabetes duration, and smoking status) [10]. They also have more frequent risk factors for PAD compared to non-Indigenous Australians, including diabetes (5–10 fold higher), [11] hypertension (3–8 fold higher), [11] and smoking (5-fold higher) [7]. Moreover, it has been estimated that the risk of major amputations (below or above the knee) in 25–49 year olds is approximately 40-fold higher in Indigenous compared to non-Indigenous Australians, attributable to the earlier onset of underlying conditions such as diabetes [7, 12]. There is, however, little understanding of the presenting characteristics and outcome of PAD in this population. Therefore, the aim of this prospective cohort study was to compare the presenting characteristics and the risk of major cardiovascular events (myocardial infarction [MI], stroke or death) in Indigenous and non-Indigenous Australians with PAD presenting to an outpatient clinic in Townsville, Queensland, Australia.

## Methods

### Study design

This was a prospective cohort study of PAD participants recruited from an outpatient clinic in Townsville Hospital (Ethics [HREC/13/QTHS/125; SSA/13/QTHS/203; HREC/14/QTHS/203; H6028 & H5537] [13, 14]. A 5-member Indigenous reference committee was consulted for approval of the research. The recruitment period for this cohort was between May 2003 and February 2016. Patients who provided written informed consent and met the following eligibility criteria were included: diagnosis of PAD by a vascular specialist as previously described, [13] including any of the following presentations: intermittent claudication, critical limb ischemia, asymptomatic or symptomatic carotid stenosis, and aortic or peripheral aneurysms. Patients who identified themselves as Aboriginal and/or Torres Strait Islanders were considered Indigenous. Patients who did not identify as Indigenous were considered non-Indigenous.

### Measures

Clinical characteristics collected for each participant included: presenting complaint, age, sex, diabetes, hypertension, smoking history, dyslipidaemia, renal function (estimated glomerular filtration rate [eGFR]), ischaemic heart disease (IHD), history of stroke, and current medications. Hypertension and diabetes were defined by history or treatment for these conditions. Cigarette smoking classification was based on smoking history, and defined as current smoker (smoked within the last month), previous smoker or never smoked. IHD was defined by a history of myocardial infarction, angina or coronary revascularization. Serum creatinine was measured using a spectrophotometry method in a pathology laboratory as previously described [1]. eGFR was calculated using the *Chronic Kidney Disease* Epidemiology Collaboration formula, as this has previously been shown to best predict complications [1]. Abdominal aortic aneurysm (AAA) was defined as an orthogonal infra-renal aortic diameter of at least 30 mm on ultrasound or computed tomography angiography [13, 14]. A significant carotid artery stenosis was defined as ≥50% using Australian Society for Ultrasound in Medicine criteria [13, 14]. Current prescriptions for aspirin, other antiplatelet agents, warfarin, beta-blockers, calcium channel blockers, angiotensin pathway inhibitors, metformin, insulin, frusemide and statins were recorded. Follow-up data was retrieved from out-patient visits, hospital chart reviews, linked data and /or inpatient admissions as previously described [13, 14]. The primary outcome was to evaluate the differences in the combined incidence of major cardiovascular events (MI, stroke or death) between Indigenous and non-Indigenous Australians with PAD. The diagnoses of MI and stroke were made by Royal Australasian College of Physicians accredited physician in accordance with international guidelines [15, 16]. Death was defined to include deaths from all causes. Linked data were based on International Classification of Diseases (ICD)-10 coding for MI (I21.0-I22.9) and stroke (I60.0-I64.0). Patients were censored at the first major event, or at the date of last review or linked data request, if they did not experience an event.

### Sample size calculation

The required sample size was estimated according to a planned Cox regression analysis, examining the association of Indigenous status with major cardiovascular events. On the basis of previous PAD studies conducted at The Townsville Hospital, the incidence of major cardiovascular events was estimated to be 30% over 3 years, [17] and the model was designed to include the following variables: age, sex, IHD, diabetes, hypertension, smoking and Indigenous status. It was therefore estimated that approximately 230 patients in total would be needed to be included in order to attain 10 outcome events per degree of freedom for each predictor variable to be included in the Cox regression analysis [18].

### Statistical analysis

The presenting symptoms and risk factors were compared between Indigenous and non-Indigenous patients using Pearson’s χ2 test and Mann Whitney U test. The combined incidence of MI, stroke or death in Indigenous and non-Indigenous groups was assessed with Kaplan-Meier analysis with differences compared using the log rank test. Multivariate Cox proportional hazards analyses were undertaken to assess the association between Indigenous status and cardiovascular events, adjusting for relevant confounding risk factors and medications (hypertension, current smoking, diabetes, IHD, age, critical limb ischemia, insulin, frusemide, angiotensin converting enzyme [ACE] inhibitor, anti-platelet or anti-coagulant prescription) across 3 different models. The selection of variables to adjust for was based on those which have been established as risk factors for PAD outcome, or were found to be significantly different between Indigenous and non-Indigenous patients. No violations of the proportional hazards assumption were observed. All analyses were performed using STATA version 14.1 (StataCorp, College Station, Texas, USA), and SPSS version 20.0 (IBM SPSS Inc., Chicago, Illinois, USA).

## Results

### Participant characteristics

Four hundred and one patients with PAD were included, of which 16 (4.0%) were Indigenous and 385 (96.0%) were non-Indigenous Australians. Indigenous participants were younger at entry (median age 63.3 [54.7–67.8] vs 69.6 [63.3–75.4], *p =* 0.005), were more likely to be current smokers (56.3% vs 31.4%, *p =* 0.041) and have insulin-treated diabetes (18.8% vs 5.2%, *p =* 0.022). Indigenous participants were also more likely to be prescribed frusemide for heart failure (Table 1). A lower proportion of Indigenous Australians were prescribed a statin, although this difference was not significant. There were no significant differences in presenting complaint at entry between Indigenous and non-Indigenous Australians (Table 2).

**Table 1:** Demographic and risk factors of included PAD patients

Characteristics	Indigenous (*n* = 16)	Non-Indigenous (*n* = 385)	*p*-value
Age (y)	63.3 (54.7–67.8)	69.6 (63.3–75.4)	**0.005**
Sex (% Males)	10 (62.5%)	293 (76.1%)	0.215
Diabetes mellitus	7 (43.8%)	103 (26.8%)	0.135
Smoker			**0.041**
Never	0 (0%)	82 (21.3%)	
Current	9 (56.3%)	121 (31.4%)	
Previous	7 (43.8%)	182 (47.3%)	
Hypertension	15 (93.8%)	290 (75.3%)	0.091
IHD	10 (62.5%)	164 (42.6%)	0.116
Stroke	1 (6.3%)	38 (9.9%)	0.632
eGFR (mL/min/1.73m^2^)	79.0 (46.5–85.8) [2]^a^	78 (60.0–91.0) [93]^a^	0.416
Medications			
Aspirin	11 (68.88%)	252 (65.5%)	0.786
Other antiplatelet	3 (18.8%)	66 (17.1%)	0.867
Frusemide	4 (25.0%)	28 (7.3%)	**0.010**
ACEI	10 (62.5%)	152 (39.5%)	0.066
Beta-Blocker	6 (37.5%)	115 (29.9%)	0.515
Calcium channel blocker	3 (18.8%)	115 (29.9%)	0.339
Metformin	4 (25.0%)	69 (17.9%)	0.472
Insulin	3 (18.8%)	20 (5.2%)	**0.022**
Statin	13 (81.3%)	273 (70.9%)	0.370

*IHD* ischaemic heart disease, *eGFR* estimated glomerular filtration rate, *ACEI* angiotensin converting enzyme inhibitor, *ARB* angiotensin receptor blocker

^a^Represents the number of missing data-points. Continuous data are presented as median [interquartile range] and were compared using Mann-Whitney U test. Nominal data are presented as number (%) and were compared using Pearson’s χ2 test. *P*-values highlighted in bold indicate significant differences

**Table 2:** Presenting complaint at entry for Indigenous and non-Indigenous patients

Characteristics	Indigenous (n = 16)	Non-Indigenous (n = 385)	*p*-value
Presentation^a^			0.385
Intermittent Claudication	8 (50.0%)	126 (32.7%)	
Critical Limb Ischemia	2 (12.5%)	24 (6.2%)	
Abdominal Aortic or peripheral aneurysm	3 (18.8%)	142 (36.9%)	
Asymptomatic carotid stenosis	2 (12.5%)	46 (11.9%)	
Symptomatic carotid stenosis	1 (6.2%)	47 (12.2%)	

^a^Indicates predominant presentation. Some patients had both lower limb symptoms and abdominal aortic aneurysm

### Association between indigenous ethnicity and major cardiovascular events

**Fig. 1:**
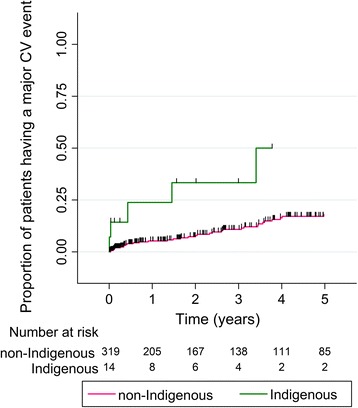
Kaplan-Meier curve illustrating the cumulative proportion of Indigenous and non-Indigenous patients with PAD, who had a major cardiovascular event (MI or stroke or death). CV, cardiovascular. Differences between both groups compared using the log-rank test (*p* = 0.004). The green line represents patients who were Indigenous, and the pink line represents non-Indigenous Australians. Vertical lines represent participants who were censored during follow-up

Median (inter-quartile range) follow-up in Indigenous patients and non-Indigenous patients was 2.5 (0.3–5.3) and 2.5 (0.4–5.4) years, respectively (*p* = 0.973). In total, five major cardiovascular events (MI, stroke, or death) were recorded in Indigenous Australians, and 45 major cardiovascular events were recorded in non-Indigenous Australians. Kaplan Meier curves illustrating the cumulative proportion of Indigenous and non-Indigenous patients who had major cardiovascular events are presented in Fig. 1. Differences between both groups were considered to be significant (log-rank test *p* = 0.004). By unadjusted Cox proportional hazard analysis, Indigenous Australians had a 3.59-fold higher risk of major cardiovascular events during follow-up compared to non-Indigenous Australians (*p* = 0.007; Table 3). By multivariate Cox proportional hazard analysis, Indigenous Australians were at ~ 5-fold greater risk of major cardiovascular events (HR 4.72 [1.41–15.78], *p* = 0.012), after adjusting for hypertension, current smoking, diabetes, IHD, age, critical limb ischemia, insulin, frusemide, ACE inhibitor, anti-platelet or anti-coagulant prescription (Table 3).

**Table 3:** Cox proportional hazard analyses for the association between Indigenous ethnicity and combined incidence of major cardiovascular events (MI, stroke or death) in PAD patients

	Unadjusted HR (95%CI)	*p*-value	Adjusted HR (95%CI)	*p*-value
MI, stroke or death				
Non-Indigenous	1.00	**–**	1.00	**–**
Indigenous	3.59 (1.42–9.07)	0.007	3.12 (1.14–8.49)	0.026 ^a^
	–	–	4.03 (1.17–13.84)	0.027 ^b^
	–	–	4.72 (1.41–15.78)	0.012 ^c^

*MI* myocardial infarction, *CI* confidence interval, *HR* hazard ratio, *IHD* ischemic heart disease, *ACE* angiotensin converting enzyme

^a^Results are adjusted for hypertension, current smoking, diabetes, IHD, and age

^b^Results are adjusted for hypertension, current smoking, diabetes, IHD, age, insulin and frusemide prescription

^c^Results are adjusted for hypertension, current smoking, diabetes, IHD, age, insulin, frusemide, ACE inhibitor, anti-platelet or anti-coagulant prescription and critical limb ischemia

## Discussion

To our knowledge, this is the first comparison of the clinical presentation and outcome of PAD in Indigenous and non-Indigenous Australians. The results of this prospective cohort study suggest that Indigenous Australians present at a younger age with PAD, have more frequent cardiovascular risk factors, and are at significantly greater risk of developing major cardiovascular events compared to non-Indigenous Australians.

In this cohort, Indigenous Australians were more likely to have insulin-treated diabetes, which is reflective of the greater severity of diabetes in this population [7]. Indigenous Australians were more likely to be prescribed frusemide, which reflects the greater burden of heart failure in this population as previously identified [19]. In terms of outcome, Indigenous Australians were at approximately 5-fold greater risk of developing cardiovascular events in comparison to non-Indigenous Australians. Notably, these differences were seen despite the Indigenous cohort being, on average, younger than the non-Indigenous cohort which suggests early onset of PAD in this population. This may be due to earlier onset of PAD risk factors, such as diabetes, in Indigenous Australians [7]. It may also reflect greater prevalence of other risk factors for PAD in this population. Indigenous Australians had significantly higher smoking rates which is consistent with large observational studies [7]. Smoking is a strong risk factor for PAD, [20] and results from the Freemantle Diabetes Study suggested that although smoking rates amongst non-Indigenous Australians has almost halved over the past 15 years, smoking rates have remained relatively unchanged in Indigenous Australians [7].

As with Australian data, previously published research in other countries focused on Indigenous populations has mainly looked at lower limb amputations secondary to diabetes-associated foot disease and cardiovascular events in patients that have diabetes [21–23]. It is therefore difficult to compare our findings to those reported regarding other Indigenous populations. In a retrospective analysis of Canadian Aboriginal and non-Aboriginal subjects with PAD, Aboriginal patients had a significantly higher prevalence of risk factors for PAD such as diabetes, hypertension, and renal failure, although Aboriginal ethnicity did not have an independent association with mortality [24]. A more comprehensive understanding of the epidemiology and outcome of PAD amongst different populations is warranted, given the high morbidity and mortality associated with this condition. In addition to differences in cardiovascular risk factors between Indigenous and non-Indigenous populations, previous studies have suggested that specific populations may have a predisposition to develop a particular anatomical distribution of arterial disease, which may contribute to poor clinical outcomes [25]. For example, in a retrospective analysis of 1215 PAD patients, the prevalence of aorto-iliac and infra-geniculate disease was higher in African-American and Asian populations compared to the Caucasian population [26–28]. In the current study, Indigenous Australians had more frequent lower limb presentations (intermittent claudication and critical limb ischemia) compared to non-Indigenous Australians, although differences were not significant. It is not yet clear whether these differences are related to ethnicity or rather clinical risk factors associated with distal PAD, such as diabetes [29].

This study has several limitations. Although our required sample size of 230 patients was exceeded, the recruited patients were unevenly distributed between the Indigenous and non-Indigenous groups (1:24). This is likely to have underpowered our study to detect significant differences in some established PAD risk factors and presenting complaints between Indigenous and non-Indigenous Australians. Although a significant association between Indigenous Australians with PAD and major cardiovascular events was established, confidence intervals were wide, which is reflective of the limited sample size, and it is possible that the true magnitude of the effect size may be different. Furthermore, data on important cardiovascular risk factors such as body mass index, glycaemic control, ankle brachial index and blood pressure were not collected. We only recruited participants from the outpatient setting, and were unable to include patients with emergency presentations, which may be of significance to Indigenous populations given greater rates and severity of diabetes and higher prevalence of complicating factors such as sepsis [30].

## Conclusion

Placing our results in the context of what was previously known, this study highlights a significant health gap for Indigenous Australian patients with PAD. Indigenous Australians presented with PAD at a younger age, and were at significantly greater risk of major cardiovascular events compared to non-Indigenous Australians. They also more commonly had cardiovascular risk factors such as current smoking and insulin-treated diabetes, emphasising the need for more intensive management of modifiable risk factors in this population. Further study in this area with larger sample sizes is required to further characterise the excess burden of PAD in the Indigenous population, which may help identify additional clinical targets for improving PAD outcome.

## Abbreviations

AAA: Abdominal aortic aneurysm
ACE: Angiotensin converting enzyme
ARB: Angiotensin converting enzyme inhibitor
CI: Confidence intervals
CV: cardiovascular
eGFR: Estimated glomerular filtration rate
HR: Hazard ratio
ICD: International Classification of Diseases
IHD: Ischaemic heart disease
MI: Myocardial infarction
PAD: Peripheral artery disease
